# Physical Activity Assessment of Physicians in Primary Healthcare Centers in Makkah, Saudi Arabia

**DOI:** 10.7759/cureus.67659

**Published:** 2024-08-24

**Authors:** Alaa G Alolayan, Salman Alsubhi

**Affiliations:** 1 Public Health, Ministry of Health, Jeddah, SAU; 2 Preventive Medicine and Public Health, Disaster Managment and Crowd Health Care, Ministry of Health, Saudi Board of Preventive Medicine, Makkah, SAU; 3 Public Health, Ministry of Health, Makkah, SAU

**Keywords:** saudi arabia, makkah, primary healthcare centers, physicians, physical activity

## Abstract

Background: Physical inactivity is a significant global health concern and a major contributor to numerous non-communicable diseases. A focus on physical activity in healthcare services, particularly among healthcare professionals, is crucial.

Objective: This study aims to explore physical activity levels and influencing factors among physicians in Primary Healthcare Centers in Makkah, Saudi Arabia.

Methods: A cross-sectional study was conducted involving physicians from Primary Healthcare Centers in Makkah. An online questionnaire was used to collect data, which included demographic factors and the General Practice Physical Activity Questionnaire (GPPAQ). Data were analyzed using the SPSS, version 29.0 (IBM Corp., Armonk, NY, USA).

Results: Of the 194 physicians included in the study, 25.3% were found to be inactive, 24.1% moderately inactive, 25.3% moderately active, and 25.3% active. There was no significant association between the Physical Activity Index and age, gender, nationality, income, having children, or job classification. However, marital status, Body Mass Index (BMI), smoking status, and the presence of diabetes mellitus, hypertension, dyslipidemia, and depression were significantly associated with the Physical Activity Index (*p*<0.05).

Conclusion: The study provides valuable insights into the physical activity levels among physicians in Makkah, Saudi Arabia. The findings suggest the need for strategies to increase physical activity among physicians, particularly those who are single, overweight or obese, smokers, and those with certain health conditions.

## Introduction

Physical inactivity has emerged as a significant issue for global health, contributing to significant morbidity and mortality worldwide. The Global Burden of Disease Study estimates that physical inactivity contributed to approximately one million deaths in 2017, which was a rise of 22% from 2007 [1]. Physical inactivity is also deemed a significant source of various non-communicable diseases (NCDs) such as ischemic stroke, hypertension, dementia, diabetes mellitus, and various types of cancers [2]. According to Santos et al., the prevalence of NCDs will reach 499.2 new cases if the current pattern of physical inactivity persists during this period. They further estimated that the economic impact of physical inactivity will reach $47.6 billion per year [3].

The World Health Organization (WHO) has launched the Global Action Plan on Physical Activity and Health from 2018-2030 which plans to lower the prevalence of physical inactivity by 15% in 2030. One aspect of WHO’s plan is to focus on physical inactivity in healthcare services [4]. The role of primary healthcare centers on population health is profound, with an impact not only on overall health but also on health equity, access to care, and health promotion [5]. Therefore, physical activity assessment at the primary healthcare center level can have a significant impact on WHO’s plan for reducing physical inactivity.

Physical activity can be stated as the movement of skeletal muscles that leads to the utilization of energy above the baseline level [6]. Exercise is a subset of physical activity that can include various activities aimed at improving the body’s fitness and overall health. Global recommendations for physical activity include participating in a minimum of 150 minutes of moderate-intensity exercise per week or 75 minutes of vigorous-intensity activity every week. However, a combination of both vigorous and moderate activities can also be incorporated to achieve equivalent health benefits [7]. In terms of metabolic equivalents (METs), a moderate physical workout relates to 3-5.9 METs whereas a high-intensity workout includes 6 or more METs. One MET is defined as an oxygen uptake of 3.5 mL/kg/min during rest [8]. Physical activity assessment in primary healthcare settings is fundamental and can have a significant impact on patient health. Stoutenberg et al. have developed an inclusive context for evaluating the impact of physical activity in healthcare systems. It comprises three steps including assessment of physical activity, physical activity counseling, and referral of patients to experts [9].

Although there has been a significant increase in physical assessment coverage in primary healthcare centers, it is still not adopted as a mandatory part of the screening process. Gonzalez-Viana et al., in their study, reported that physical inactivity screening was 14.4% in 2008 and increased to 69.6% in 2015 [10]. Similarly, a cross-sectional study by Samir et al. conveyed that the prevalence of physical inactivity was 72.6% among obese attendants in a healthcare center in Pakistan [11]. Based on the analysis of 1.9 million contestants, the global prevalence of physical inactivity was reported at 27.5% in 2016 [12]. According to a systemic review from Saudi Arabia, physical inactivity ranged from 26% to 85% among males whereas 43% to 91% of females were physically inactive [13]. Therefore, creating a list of physical activities could increase the awareness level of both physicians and patients regarding this condition. This can further help physicians formulate strategies for improving patient outcomes. Furthermore, physical activity can also be incorporated into electronic medical record (EMR) systems [14]. American Heart Association has also encouraged the routine assessment of physical activity in healthcare sites [15]. 

Given the rising global physical inactivity and health risks, it's crucial to comprehend physical activity patterns, particularly among healthcare professionals. In Saudi Arabia, high levels of inactivity can impact public health negatively. This study aims to assess physical activity levels and investigate the demographic and health-related factors influencing these levels among physicians in Primary Healthcare Centers in Makkah, Saudi Arabia.

## Materials and methods

The study was conducted in Makkah City, Saudi Arabia. Data collection took place from April 2024 to May 2024. The focus of the study was on physicians working in these centers, given the potential influence of their personal physical activity levels on their health outcomes and professional performance.

The study population comprised physicians from Makkah City's Primary Healthcare Centers. A sample size of 194 was estimated using an online sample size calculator, taking into account the total number of physicians in Makkah Primary Healthcare Centers (390), a 5% margin of error, a 95% confidence level, and a 50% response distribution.

The eligibility criteria for this study included primary healthcare physicians, defined as medical doctors who provide first-contact and continuous care for patients at Primary Healthcare Centers, working in Makkah City, Saudi Arabia, who are willing to participate in the study. Physicians working in other specialties were excluded from this study. A convenient sampling technique was employed to recruit the participants from three different Primary Healthcare Centers in Makkah: Security Forces Clinic, Aliskan Primary Healthcare, and Batha Quraish Primary Healthcare. Data were collected using an online questionnaire distributed to the physicians.

The online questionnaire consisted of two sections: demographic characteristics and the General Practice Physical Activity Questionnaire (GPPAQ) (Appendix 1). The GPPAQ, a validated instrument developed by the London School of Hygiene and Tropical Medicine, was used to measure the physical activity levels of individuals. No missing data were present as all questions were required.

The GPPAQ includes items that assess various aspects of physical activity: work-related physical activity, travel to and from work, recreational physical activity, household activities, gardening and DIY activities, and walking pace. Questions cover the type of physical activity performed during work hours, the mode of transportation used for commuting, physical activities during leisure time such as exercise and sports, activities related to household chores and childcare, gardening and DIY tasks, and the usual pace of walking. The responses to these items help categorize individuals into different levels of physical activity, ranging from inactive to active.

Data were analyzed using SPSS version 29.0 (IBM Corp., Armonk, NY, USA). Proportions and frequencies were used to describe and summarize categorical variables. The Chi-Square test and Fisher Freeman-Halton Exact test were used for the bivariate analysis of categorical outcomes. P-values less than 0.05 were considered significant, with a 95% confidence level used to make inferences. Ethical approval was secured from the Institutional Review Board (IRB) at the Security Forces Hospital Program in Holy Capital (IRB number: 0709-210424) prior to data collection. All data were handled with utmost confidentiality, stored securely, and used exclusively for research purposes.

## Results

A total of 194 physicians qualified and were included in the study analysis. The demographic characteristics of the participants are given in Table 1. The participants' ages ranged from 26 to 52 years, with a median age of 35 years. The majority of participants were under 45 years of age 184 (94.8%), indicating a predominantly young population. The proportion of females was somewhat higher 103 (53.1%) compared to males 91 (46.9%). The vast majority were Saudi nationals 183 (94.3%), and most of the participants earned ≤20,000 Saudi Riyals per month. The majority of the participants were married 135 (69.6%) and had children 117 (60.3%).

**Table 1:** Demographic characteristics This table presents the demographic characteristics of the 194 physicians included in the study, including age, gender, nationality, marital status, income, having children, and job classification.

Variable	n=194		N	%
Age		≤35	105	54.1%
		36 – 45	79	40.7%
		>45	10	5.2%
Gender		Male	91	46.9%
		Female	103	53.1%
Nationality		Saudi	183	94.3%
		Non-Saudi	11	5.7%
Marital Status		Single	59	30.4%
		Married	135	69.6%
Income (Saudi Riyals)		≤20,000	113	58.2%
		>20,000	81	41.8%
Do you have children?		Yes	117	60.3%
		No	77	39.7%
Job classification		General practitioner	41	21.1%
		Resident	64	33.0%
		Registrar	24	12.4%
		Consultant	65	33.5%

Table 2 provides an indication of the health determinants of the participants. Over half of the participants were overweight 105 (54.1%), while 70 (36.1%) had a healthy body mass index (BMI), and 19 (9.8%) were obese. A quarter of the participants were smokers (49; 25.3%), and various health conditions such as diabetes mellitus, hypertension, dyslipidemia, depression, and thyroid disease were present in a small proportion of the participants.

**Table 2:** Health determinants This table presents the health-related characteristics of the 194 physicians, including Body Mass Index (BMI), smoking status, and the prevalence of various health conditions such as diabetes mellitus, hypertension, dyslipidemia, depression, and thyroid disease.

Variable	n=194		N	%
Body Mass Index		Healthy	70	36.1%
		Overweight	105	54.1%
		Obese	19	9.8%
Smoking		Yes	49	25.3%
Diabetes Mellitus		Yes	11	5.7%
Hypertension		Yes	24	12.4%
Dyslipidemia		Yes	25	12.9%
Depression		Yes	12	6.2%
Thyroid disease		Yes	18	9.3%

Table 3 outlines the physical activity levels of the participants. The Physical Activity Index was evenly distributed among the participants, with 49 (25.3%) being inactive, 47 (24.1%) moderately inactive, 49 (25.3%) moderately active, and 49 (25.3%) active. Additionally, when it comes to walking, 117 (60.3%) of participants presented less than an hour of walking in the last week, and 11 (5.7%) walked for three hours or more.

**Table 3:** Physical activity among participants This table outlines the physical activity levels of the participants, including the Physical Activity Index, walking hours during the last week, housework/childcare hours, gardening/DIY activities hours, and walking pace.

Variable	n=194		N	%
Physical Activity Index		Inactive	49	25.3%
		Moderately inactive	47	24.1%
		Moderately active	49	25.3%
		Active	49	25.3%
Walking (hours during last week)		None	16	8.2%
		<1	101	52.1%
		More than 1 but <3	66	34.0%
		≥3	11	5.7%
Housework/Childcare (hours during last week)		None	36	18.6%
		<1	84	43.3%
		More than 1 but <3	52	26.8%
		≥3	22	11.3%
Gardening/Do It Yourself activities (hours during last week)		None	47	24.2%
		<1	78	40.2%
		More than 1 but <3	49	25.3%
		≥3	20	10.3%
Walking pace		Slow pace	46	23.7%
		Steady average pace	104	53.6%
		Brisk pace	28	14.4%
		Fast pace	16	8.3%

Table 4 presents the association between demographic factors and the Physical Activity Index. No significant association was found between the Physical Activity Index and age, gender, nationality, income, having children, or job classification. However, marital status was significantly associated with the Physical Activity Index, with married individuals more likely to be moderately active or active (*p*=0.039).

**Table 4:** Association between demographic factors and Physical Activity Index Chi-Square test, Fisher Freeman-Halton Exact test. *. Association is significant at p-value <0.05

Variable	n=194		Physical Activity Index									
			Inactive (n=49)		Moderately inactive (n=47)		Moderately active (n=49)		Active (n=49)			
			N	%	N	%	N	%	N	%	p-value	x^2^
Age		≤35	25	51.0%	25	53.2%	28	57.1%	27	55.1%	0.985	1.242
		36 - 45	22	44.9%	19	40.4%	18	36.7%	20	40.8%		
		>45	2	4.1%	3	6.4%	3	6.2%	2	4.1%		
Gender		Male	22	44.9%	23	48.9%	18	36.7%	28	57.1%	0.242	4.254
		Female	27	55.1%	24	51.1%	31	63.3%	21	42.9%		
Nationality		Saudi	43	87.8%	45	95.7%	48	98.0%	47	95.9%	0.230	4.523
		Non-Saudi	6	12.2%	2	4.3%	1	2.0%	2	4.1%		
Marital Status		Single	17	34.7%	20	42.6%	14	28.6%	8	16.3%	0.039*	8.370
		Married	32	65.3%	27	57.4%	35	71.4%	41	83.7%		
Income (Saudi Riyals)		≤20,000	30	61.2%	26	55.3%	29	59.2%	28	57.1%	0.961	.387
		>20,000	19	38.8%	21	44.7%	20	40.8%	21	42.9%		
Do you have children?		Yes	25	51.0%	27	57.4%	30	61.2%	35	71.4%	0.216	4.475
		No	24	49.0%	20	42.6%	19	38.8%	14	28.6%		
Job classification		General practitioner	13	26.5%	13	27.7%	7	14.3%	8	16.3%	0.681	6.577
		Resident	17	34.7%	13	27.7%	16	32.7%	18	36.7%		
		Registrar	5	10.2%	4	8.5%	9	18.3%	6	12.3%		
		Consultant	14	28.6%	17	36.2%	17	34.7%	17	34.7%		

Table 5 depicts the association between health determinants and the Physical Activity Index. Significant associations were found between the Physical Activity Index and BMI, smoking status, and the presence of diabetes mellitus, hypertension, dyslipidemia, and depression. For example, those with a healthy BMI were more likely to be moderately active or active (*p*=0.002), and smokers were less likely to be moderately active or active (*p*=0.049). Similarly, those with diabetes mellitus, hypertension, dyslipidemia, or depression were less likely to be moderately active or active (*p*<0.05).

**Table 5:** Association between health determinants and Physical Activity Index Chi-Square test, Fisher Freeman-Halton Exact test. *. Association is significant at p-value <0.05

Variable	n=194		Physical Activity Index									
			Inactive (n=49)		Moderately inactive (n=47)		Moderately active (n=49)		Active (n=49)			
			N	%	N	%	N	%	N	%	p-value	x^2^
Body Mass Index		Healthy	11	22.4%	14	29.8%	23	46.9%	22	44.9%	0.002*	18.697
		Overweight	28	57.2%	27	57.4%	23	46.9%	27	55.1%		
		Obese	10	20.4%	6	12.8%	3	6.2%	0	0.0%		
Smoking		Yes	18	36.7%	12	25.5%	6	12.2%	13	26.5%	0.049*	7.858
		No	31	63.3%	35	74.5%	43	87.8%	36	73.5%		
Diabetes Mellitus		Yes	7	14.3%	3	6.4%	0	0.0%	1	2.0%	0.007*	9.616
		No	42	85.7%	44	93.6%	49	100.0%	48	98.0%		
Hypertension		Yes	12	24.5%	8	17.0%	1	2.0%	3	6.1%	0.002*	14.164
		No	37	75.5%	39	83.0%	48	98.0%	46	93.9%		
Dyslipidemia		Yes	12	24.5%	8	17.0%	3	6.1%	2	4.1%	0.007*	11.973
		No	37	75.5%	39	83.0%	46	93.9%	47	95.9%		
Depression		Yes	5	10.2%	6	12.8%	0	0.0%	1	2.0%	0.012*	9.445
		No	44	89.8%	41	87.2%	49	100.0%	48	98.0%		
Thyroid disease		Yes	7	14.3%	4	8.5%	5	10.2%	2	4.1%	0.371	3.096
		No	42	85.7%	43	91.5%	44	89.8%	47	95.9%		

## Discussion

The findings of this study provide insight into the level of physical activity of the physicians, in the Makkah region of Saudi Arabia. This study found that a low number of physicians were moderately active (25.3%) or active (25.5%), whereas 25.3% were inactive. Previously various studies from the Makkah region have reported the physical activity level of health professionals. The results of the present study were comparable to the findings of Melebari and Khan [16]. Their study included 196 physicians from the primary healthcare centers in Maakah. Their findings revealed that about 45.9% of the physicians were overweight. Furthermore, 71.9% of the participants did not perform any vigorous activity in the last seven days, whereas 30.6% of physicians reported being moderately active [16]. Similar findings have been reported by Ageel who found that 48.3% of family medicine trainees had a low level of physical activity whereas only 11.7% had an optimum level of physical activity [17]. 

The results of the present study align with other studies performed in the region. For example, a study conducted in Bahrain found that 72.5% of physicians had an above-normal BMI, and only 29.6% of the participants reported performing ≥ 30 min of exercise per week [18]. Similarly, a study from Qatar reported that only 39.5% of primary healthcare physicians were physically active, as per the recommendation of the WHO. Furthermore, the study also reported that only 60% of the patients were counseled about physical activity; however, they found no association between the physician’s physical activity and the number of patients counseled [19]. The low physical activity identified in this study resonated with the general practice of the public regarding physical activity. According to WHO, more than 30% of the world’s population does not attain the minimum suggested physical activity level [16]. The low activity levels among physicians can be due to long working hours, with prolonged inactivity periods.

The findings of the present study are contrasted by another study from Jeddah, Saudi Arabia. Khateeb et al. surveyed 178 physicians practicing at King Abdul Aziz Hospital in Jeddah. More than half (74.6%) of the physicians reported regular physical activity. Among 129 doctors who stated low to high physical activity, it was found that 50% were in the moderate to high category of physical activity and only 23.7% were in the low category [20]. Another study enrolled primary health care physicians working in the primary care hospitals in two cities of the Al-Jouf region. This study found that 65.2% of the physicians were involved in moderate to strong physical activity. About 34.8% of the physicians were found to be physically inactive. This study found that neither location, nationality nor gender was significantly associated with physical activity. Furthermore, physically active physicians were more involved in guiding their patients regarding physical activity compared to less physically active physicians [21]. 

In this study, while no association was found between physical activity and demographic parameters, marital status was significantly associated with physical activity levels, with married individuals more likely to be moderately active or active. This is different from what has been reported in the literature previously. Generally, physical activity is higher in single individuals compared to married counterparts [22]. Similarly, a negative association was seen between smoking and physical activity levels. A significant body of evidence resonates with these findings as smoking is a well-established contributor to low physical activity [23-25]. Similarly, a negative relationship was also reported between physical activity and diabetes mellitus, hypertension, dyslipidemia, or depression. Despite the fact that physical activity improves diabetes and dyslipidemia symptoms, a low level of physical activity has been reported among diabetic patients [26]. Similarly, a significant association between physical activity and chronic diseases has also been reported by Crnkovic et al. [27].

It is evident from the present study and previous studies that the prevalence of physical activity among the physicians of Makkah City is comparably low. The low prevalence is thought to be associated with perceived barriers and sedentary lifestyles. The main barriers as reported by the physicians are shortage of time, lack of physical activity partners, and motivation [28]. Among all, lack of time is the most significant obstacle [29]. The Ministry of Health (MOH) of Saudi Arabia has launched various physical activity initiatives. One such step includes building more gyms that can operate for 24 hours. This can be helpful for physicians to perform some physical activities according to their work schedules and time [16].

Recommendations

This study recommends promoting daily physical activities, like walking or cycling to work, among physicians in Makkah region. Establishing wellness programs within healthcare centers that encourage regular exercise could be beneficial. Also, physicians should be educated on the benefits of physical activity and strategies to overcome barriers to exercise. Collaborations with local fitness centers for discounted memberships could be explored. Regular monitoring of physicians' physical activity levels is crucial to assess the effectiveness of these interventions. Improving physical activity among physicians could enhance their health, professional performance, and the health advice they provide to their patients.

Strengths

The strengths of this study include the utilization of a validated instrument (General Practice Physical Activity Questionnaire, GPPAQ) for assessing physical activity levels, which lends credibility to the results. Furthermore, the study recruited participants from three different primary healthcare centers in Makkah City, ensuring a diverse and representative sample of the physician population. The relatively large sample size, compared to similar studies, enhances the reliability and generalizability of the findings. Additionally, the use of robust statistical methods, including the Chi-Square test and Fisher Freeman-Halton Exact test, provided precise analysis and interpretation of the data, thereby strengthening the study's conclusions.

Limitations

However, there were some limitations. The data was collected through self-reported questionnaires, which can be biased as participants may overestimate or underestimate their physical activity levels. To address this in future studies, it would be beneficial to use objective measures of physical activity, such as wearable fitness trackers. The study was cross-sectional, limiting the ability to determine causality. Future research could use a longitudinal design to better assess causal relationships. The study was conducted only in primary healthcare centers in Makkah, restricting the generalizability of the results to other regions or healthcare settings. Expanding the study to include multiple regions and diverse healthcare settings would improve generalizability. Finally, the study did not consider the possible influence of cultural or societal factors on physical activity levels. Including qualitative methods or additional surveys to explore these influences could provide a more comprehensive understanding.

## Conclusions

In conclusion, this study provides valuable insights into the physical activity levels of physicians in Makkah region of Saudi Arabia. The research revealed a significant proportion of physicians exhibited low levels of physical activity. The study also found associations between physical activity and factors such as marital status, smoking, and the presence of chronic diseases such as diabetes mellitus, hypertension, dyslipidemia, and depression. These findings highlight the importance of encouraging physical activity among physicians. This could improve their personal health, professional performance, and patient advice. Further research is needed to identify strategies for promoting activity, considering barriers like time constraints and lack of motivation.

## Appendices

General Practice Physical Activity Questionnaire

**Table 6:** The General Practice Physical Activity Questionnaire (GPPAQ) The General Practice Physical Activity Questionnaire (GPPAQ) is an open access tool developed by the London School of Hygiene and Tropical Medicine authored by the Physical Activity Policy, Health Improvement Directorate, National Health Service, The United Kingdom.

1	Please tell us the type and amount of physical activity involved in your work. ”only one choice				Please mark one box only	
a	I spend most of my time at work sitting (such as in an office)					
b	I spend most of my time at work standing or walking. However, my work does not require much intense physical effort					
2	During the last week, how many hours did you spend on each of the following activities	None	Some but less than 1 hour		1 hour but less than 3 hour	3 hours or more
a	Physical exercise such as swimming, jogging, aerobics, football, tennis, gym workout etc.					
b	Cycling, including cycling to work and during leisure time					
c	Walking, including walking to work, shopping, for pleasure					
d	Housework/Childcare					
e	Gardening/ Diy					
3	How would you describe your usual walking pace?	Slow pace (i.e. less than 3 mph)		Steady average pace	Brisk pace	Fast pace(i.e. over 4mph)
